# Severe refeeding syndrome after starvation ketoacidosis requiring stopping feeds

**DOI:** 10.5414/CNCS111119

**Published:** 2023-06-29

**Authors:** Bana Hadid, Farid Arman, Shayan Shirazian

**Affiliations:** 1 State University of New York Downstate Health Sciences University, College of Medicine, Brooklyn, and; 2 Columbia University, Department of Nephrology, NY, USA

**Keywords:** starvation ketoacidosis, refeeding syndrome, NPO, stop feeds, electrolyte imbalance

## Abstract

Introduction: Starvation ketoacidosis (SKA) is a rare cause of ketoacidosis in the general population but can be seen with malignancy. Patients often respond well to treatment, but some rarely develop refeeding syndrome (RFS) as their electrolytes drop to dangerous levels causing organ failure. Typically, RFS can be managed with low-calorie feeds, but sometimes patients require a halt in feeds until their electrolyte imbalances are managed. Case report: We discuss a woman with synovial sarcoma on chemotherapy who was diagnosed with SKA and then developed severe RFS after treatment with intravenous dextrose. Phosphorus, potassium, and magnesium levels dropped precipitously and remained fluctuant for 6 days. She also developed normal sinus ventricular tachycardia, premature ventricular beats, and bigeminy. She could not tolerate calorie supplementation at that time. She was managed with electrolyte repletions until clinically stable and then progressed to a liquid diet. Discussion: We present a unique case of severe SKA that resulted in RFS requiring nihil per orem (NPO) treatment for 6 days. There are no specific guidelines for SKA or RFS management. Patients with pH < 7.3 may benefit from baseline serum phosphorus, potassium, and magnesium levels. Clinical trials are needed to further study which patients may benefit from starting at a low-calorie intake versus those that require holding nutrition until clinically stable. Conclusion: Completely stopping caloric intake until a patient’s electrolyte imbalance improves is an important management aspect of RFS to underscore and study, as grave complications can occur even with cautious refeeding regimens.

## Introduction 

Starvation ketoacidosis (SKA) is an uncommon cause of ketoacidosis, a metabolic state associated with pathologically high ketone body concentrations in serum and urine [1]. Blood glucose is depleted during periods of fasting, and insulin levels decline, stimulating gluconeogenesis and glycogenolysis, and eventually the formation of acidic ketone bodies [1]. Acidosis is typically mild (pH > 7.3) [2], however physiologic stress can worsen the acidosis [3]. Treatment is aimed at stimulating endogenous insulin through calorie infusion. However, this increases the risk of refeeding syndrome (RFS) occurring within the first 3 – 5 days of re-initiating feeds in malnourished patients. In RFS, there is massive insulin release leading to potentially drastic shifts in fluids and electrolytes which can cause further organ dysfunction. The hallmark feature is hypophosphatemia; however, hypokalemia, hypomagnesemia, decreased thiamine, and increased sodium and water retention can also be seen [4]. Hypophosphatemia, hypokalemia, and hypomagnesemia can precipitate cardiac arrhythmias due to disruption in membrane concentration gradients. Thiamine deficiency can lead to wet and dry beriberi syndrome. Sodium retention can lead to fluid overload. To try to prevent complications, most studies in the literature recommend starting patients on low-calorie diets and advancing gradually. Here, we discuss a unique case of grave RFS in the setting of SKA. A patient with elbow joint synovial sarcoma presented with significant acidemia (pH < 7.3) secondary to starvation. Furthermore, she developed severe RFS where her electrolytes decreased by > 30% and she experienced cardiac arrhythmias all within 5 days of reinitiating energy provision [5]. Additionally, managing her RFS proved difficult as she could not tolerate any calorie supplementation for 6 days. She was treated with electrolyte repletions until clinically stable and then progressed to a liquid diet. 

## Case report 

A 44-year-old woman with a history of left elbow synovial sarcoma who has had multiple rounds of chemotherapy presented to the hospital with poor oral intake associated with severe nausea and vomiting for ~ 1 month. The week prior to presentation, she was vomiting up to 3 times a day and could not tolerate any food or medication. On review of systems, she endorsed a fever of up to 38.33 °C (101 °F) measured at home, chills, and dysuria. She denied hematemesis. She was recently treated for *Escherichia coli* urosepsis. She had multiple hospitalizations in the past year for complications secondary to chemotherapy such as intractable vomiting and aspiration pneumonia. The patient denied any alcohol use, drug use, or ingestion of any toxic substances. 

The patient was tachycardic (130 beats per minute), in sinus rhythm on electrocardiogram (EKG), tachypneic (25 breaths per minute), and afebrile. Blood pressure and oxygen saturation on room air were within normal limits. She weighed 56.7 kg, was 157.5 cm tall, and had a body mass index of 22.9 kg/m^2^. On physical exam, the patient appeared uncomfortable in pain but was alert and oriented. Her mucous membranes were dry. The abdomen was diffusely tender to palpation with no rigidity or guarding. The remainder of the exam was unremarkable. 

Laboratory values are summarized in Table 1. Initial venous blood gas showed a pH of 7.14, partial pressure of CO_2_ of 23 mmHg, and bicarbonate of 8 mmol/L. Other remarkable values included serum sodium 131 mmol/L, potassium 3.1 mmol/L, blood urea nitrogen 9 mg/dL, creatinine 1.16 mg/dL, glucose 58 mg/dL, measured serum osmolality 277 mOsm/kg, and albumin 4.4 g/dL. The anion gap was 25 (24 when corrected for albumin). The osmolal gap was 9.0 mOsm/kg. Further investigation revealed serum calcium 11.9 mg/dL, magnesium 2.0 mg/dL, lactic acid 1.3 mmol/L, β-hydroxybutyrate (BHB) 6.81 mmol/L (normal range < 0.6 mmol/L), acetaminophen < 5.0 µg/mL, and salicylate < 0.3 mmol/L. Urinalysis revealed ketonuria ≥ 150 mmol/L. Chest X-ray was unrevealing. 

The patient had a high anion gap metabolic acidosis (HAGMA) with appropriate respiratory compensation. She had ketosis, hypokalemia, and hypoglycemia. Studies showed negative osmolal gap, urinary sepsis workup, lactic acid, salicylate levels, and acetaminophen levels. The laboratory and history findings combined suggested a diagnosis of SKA. She was given a dextrose 5% solution, intravenous fluids (IVFs), and potassium repletion of 120 mEq. Laboratory investigation 1 day later revealed a pH of 7.41, partial pressure of CO_2_ of 29 mmHg, and anion gap 17, in addition to serum bicarbonate of 17 mmol/L, BHB 0.77 mmol/L, potassium < 1.5 mmol/L, magnesium 0.7 mg/dL, and phosphorus < 0.6 mg/dL. She developed frequent ventricular arrhythmias (premature ventricular beats, bigeminy, and normal sinus ventricular tachycardia). Her peak creatinine was 1.37 mg/dL, and levels ranged from 0.88 to 1.37 mg/dL during her course. 

**Figure 1.**
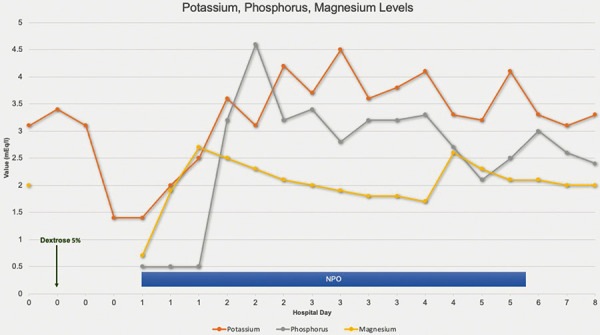
Potassium, phosphorus, and magnesium levels.

Given that serum chemistries decreased by more than 30% (~ 60% decrease in potassium, ~ 65% decrease in magnesium) within 24 hours of refeeding and that she developed organ dysfunction, the patient was subsequently diagnosed with severe RFS [5]. She was given phosphorus, potassium, and magnesium supplementation. Still, her electrolytes did not stabilize at acceptable levels (Figure 1). Because of this, she was kept nihil per orem (NPO) for 6 days to prevent exacerbating her RFS. Overall, the patient received 590 mEq potassium, 2,000 mEq-1,280 mEq-2,240 mEq total of phosphorus-sodium-potassium packets, respectively, 15 mmol of IV phosphorus, and 4 g of IV magnesium over the 6 days she was NPO. When she improved clinically and her electrolytes stabilized, caloric intake was gradually introduced and increased from a clear liquid diet to a thin liquid diet as tolerated. She was discharged after 8 days. Laboratory levels on the day of discharge showed phosphorus 2.4 mEq/L, potassium 3.3 mEq/L, and magnesium 2.0 mEq/L. 

## Discussion 

This study reports on a case of severe RFS in the setting of SKA. RFS is a rare complication of reintroducing and/or increasing calories after a period of decreased or absent caloric intake such as SKA, but few case reports on SKA mention it as a sequela [6, 7, 8, 9, 10]. To the best of our knowledge, there is only one other case reported of RFS in the setting of SKA [9]. 

Boal et al. [9] describe a case similar to ours of a 66-year-old man diagnosed with SKA who developed RFS after the introduction of IVFs and dextrose solution. The patient presented with an anion gap of > 35.7 mEq/L and a pH of 7.13. Akin to our patient, theirs was found to have a physiological stress that likely exacerbated his acidosis, acute cholecystitis due to predominantly Gram-negative bacteria and possible pancreatitis. Boal et al.’s [9] patient also developed severe RFS [5]. However, unlike our case, the authors reported being able to manage their patient in a fairly straightforward manner, with reduced caloric supplementation and a gradual increase. In comparison, we had to halt our patient’s caloric supplementation for almost a week. 

The pathophysiology of SKA and RFS is well described [5, 10, 11]. As the fasting state progresses, there is decreasing insulin and increasing glucagon, epinephrine, and cortisol. The body shifts from glycolysis to gluconeogenesis, lipolysis, and ketogenesis. Hepatic glycogen stores are depleted. Triglycerides are broken down to free fatty acids, which are then oxidized to acidic ketone bodies, including BHB [12]. Dangerous acidosis can occur in states of stress where there are increased catecholamines and cortisol which further perpetuate lipolysis, hyperketonemia, and catabolism [13]. This can cause total body deficits in fats, proteins, glucose, vitamins, and electrolytes like phosphate, potassium, magnesium, and thiamine. When reintroducing carbohydrates after starvation, there is an increase in insulin that causes an intracellular shift and utilization of the already low total body phosphate, potassium, magnesium, and thiamine. Insulin drives phosphorus intracellularly through phosphorylation of glucose as glycolysis is initiated; it also stimulates the sodium-potassium pump, driving potassium into cells. The mechanism for magnesium depletion is not well understood. RFS occurs when there is severe depletion of serum electrolytes and vitamins. The hallmark is hypophosphatemia, which leads to widespread dysfunction of cellular processes as adenosine triphosphate, the main energy source in humans, is exhausted [4, 5]. This can lead to decreased cardiac contractility and respiratory muscle dysfunction progressing to acute cardiopulmonary failure and death. Hypophosphatemia can also decrease the production of 2,3-bisphosphoglycerate resulting in tissue hypoxia which can result in worsening acidosis. 

Incidence of RFS has been reported to range from 2 to 34%, but the fact that there is no universally agreed-upon definition of RFS makes studying it difficult [14, 15, 16]. Incidence varies per patient population (e.g., 14% of geriatric patients, 25% of patients with cancer, and 28% of patients with anorexia nervosa) [11] but all share a common theme of prolonged undernourishment and ongoing electrolyte loss [5, 17]. Mortality can reach up to 71%, and patients with RFS have longer hospital stays [16]. 

While there is no single “best” screening tool, the Britain’s National Institute for Health and Care Excellence (NICE) guidelines, published in 2006, are commonly used [5, 11, 16, 17]. It is worth noting that in two studies, NICE guidelines had low sensitivity [18, 19]. NICE criteria use at least 1 – 2 of the following: BMI < 18.5 kg/m^2^, unintentional weight loss > 10 – 15% within 3 – 6 months, little or no nutritional intake for > 5 – 10 days, a history of alcohol or drug use (including insulin, chemotherapy, antacids, or diuretics), or low serum levels of potassium, phosphate, or magnesium before feeding [20]. 

A recent observational, retrospective cohort study published in 2022 by Liu et al. [21] compared various screening tools, including the American Society for Parenteral and Enteral Nutrition (ASPEN) and modified NICE, and found that the ASPEN criteria had the highest sensitivity and area under the receiver operating characteristic curve at 53.6% and 0.597, respectively. Their guidelines were published in 2020 when an interdisciplinary group worked on consolidating the heterogeneous literature surrounding RFS into a unified clinical definition as well as an updated risk assessment tool. They defined RFS as: 1) a decrease in serum phosphorus, potassium, and/or magnesium levels by 10 – 20% (mild RFS), 20 – 30% (moderate RFS), or > 30% and/or organ dysfunction resulting from a decrease in any of these and/or due to thiamine deficiency (severe RFS) and 2) occurring within 5 days of reinitiating or substantially increasing energy provision [5]. They also created a new risk assessment tool that built on the NICE criteria and added physical exam findings including loss of subcutaneous fat and muscle mass. ASPEN divided patients into moderate or significant risk for RFS. They did not create a mild criterion as they thought it would make the definition too sensitive in addition to being clinically insignificant [5]. Using these criteria, our patient had a significant risk for RFS, and her case was severe [5]. 

There is no universal recommendation for how to reintroduce feeds and manage RFS. We followed the NICE criteria, which recommended starting nutrition support of 5 – 10 kcal/kg/day (5 kcal/kg/day in “extreme” cases of BMI < 14 kg/m^2^ or negligible food intake for > 15 days). Since our patient’s BMI was > 14 kg/m^2^ and she presented with only 1 week of negligible food intake, she received ~ 400 calories in the form of IV dextrose 5% and sodium chloride 0.9% solution. When our patient developed severe RFS, we decided to stop nutritional support, and keep her NPO while repleting her electrolytes. After 6 days, her serum electrolytes stabilized and she appeared improved clinically; thus, a trial of liquid oral supplementation was started in addition to electrolyte replacement. She tolerated liquid feeds well. 

Of note, our patient did not have baseline serum phosphorus levels, and her serum magnesium levels were not measured regularly. As hypophosphatemia is the hallmark of RFS, it is helpful to have those levels available in patients at high risk of RFS. As such, we propose measuring baseline serum phosphorus, magnesium, and potassium levels in patients with malnutrition, with little to no oral intake for ≥ 7 days, and in groups with increased risk of RFS. More robust clinical trials are needed to elucidate electrolyte thresholds that are safe to begin feeds immediately versus those that need replacement prior to administering nutritional support. 

Many recommendations suggest starting at a low-calorie diet (ranging from 5 to 25 kcal/kg/day) and advancing slowly [11, 17, 20], and only a few explicitly stated holding nutrition until a patient is clinically stable with normalized electrolyte levels [5, 22, 23]. Chowdhary et al. [24] reported two cases of RFS in patients on hemodialysis whose enteric nutrition was interrupted while the patients’ dyselectrolytemia and organ dysfunctions were managed. In this study, our patient’s RFS was so severe and difficult to manage that she could not be given oral or parental nutrition for 6 days. Completely stopping caloric intake until a patient’s electrolyte imbalance and clinical status improves is an important management aspect of RFS to underscore, as grave complications can occur even with cautious refeeding regimens [25]. 

## Conclusion 

Patients with SKA with pH < 7.3 can be at increased risk for RFS. Additional risk factors include patients with malnutrition, with little to no oral intake for ≥ 7 days, and/or in groups with prolonged undernourishment and ongoing electrolyte loss. Regarding RFS management, many guidelines recommend starting at 5 – 25 kcal/kg/day and advancing slowly. Few explicitly mention halting caloric feeds completely in patients with severe RFS. Continuing a low-calorie diet when a patient’s potassium, phosphorus, and/or magnesium are not well controlled can have detrimental effects. As there is little data on when to start with low-calorie feeds and when to halt them, we believe this is an important topic for future studies. It may be beneficial to measure baseline serum phosphorus, magnesium, and potassium levels in these patients to further elucidate electrolyte thresholds that should be considered for refeeding. 

## Funding 

This study was not funded by any grant funding or industry support. 

## Conflict of interest 

None. 

**Table 1.** Serum laboratory values.

	Reference range	Day 0	Day 1	Day 8
pH	7.35 – 7.45	7.14	7.41	
pCO_2_ (mmHg)	35 – 45 mmHg	23	29	
Sodium (mmol/L)	137 – 145 mmol/L	131	142	
Potassium (mmol/L)	3.5 – 5.1 mmol/L	3.1	< 1.5	3.3
Chloride (mmol/L)	98 – 107 mmol/L	98	108	
Bicarbonate (mmol/L)	19 – 27 mmol/L	8	17	
Blood urea nitrogen (mg/dL)	7 – 26 mg/dL	9	3	
Creatinine (mg/dL)	0.50 – 0.95 mg/dL	1.16	1.25	
Glucose (mg/dL)	75 – 100 mg/dL	58	> 1,500	
Anion gap	5 – 17	25	17	
Serum osmolality (mOsm/kg)	275 – 295 mOsm/kg	277	N/A	
Calcium (mg/dL)	8.8 – 10.3 mg/dL	11.9	10.2	
Magnesium (mg/dL)	1.6 – 2.6 mg/dL	2.0	0.7	2.0
Phosphorus (mg/dL)	2.5 – 4.5 mg/dL	N/A	< 0.6	2.4
β-hydroxybutyrate (mmol/L)	< 0.6 mmol/L	6.81	0.77	
Acetaminophen (µg/mL)	10 – 20 µg/mL	< 5.0	N/A	
Salicylate (mmol/L)	< 2.2 mmol/L	< 0.3	N/A	
Urinalysis ketones (mmol/L)	< 0.6 mmol/L	≥ 150		
Lactic acid (mmol/L)	0.5 – 2.2 mmol/L	1.3		
Protein (g/dL)	6.0 – 8.3 g/dL	7.2		
Albumin (g/dL)	3.5 – 5.5 g/dL	4.4		
Hemoglobin (g/dL)	12.1 – 15.1 g/dL	12.8		
White blood cell (K/µL)	5 – 10 K/µL	7.08		
Platelet (10^9^/L)	150 – 400 10^9^/L	297
